# Identifying Myocardial Infarction Using Hierarchical Template Matching–Based Myocardial Strain: Algorithm Development and Usability Study

**DOI:** 10.2196/22164

**Published:** 2021-02-10

**Authors:** Jayendra Maganbhai Bhalodiya, Arnab Palit, Gerard Giblin, Manoj Kumar Tiwari, Sanjay K Prasad, Sunil K Bhudia, Theodoros N Arvanitis, Mark A Williams

**Affiliations:** 1 Institute of Digital Healthcare Warwick Manufacturing Group University of Warwick Coventry United Kingdom; 2 Warwick Manufacturing Group University of Warwick Coventry United Kingdom; 3 Royal Brompton and Harefield NHS Foundation Trust London United Kingdom; 4 National Institute of Industrial Engineering Mumbai India

**Keywords:** left ventricle, myocardial infarction, myocardium, strain

## Abstract

**Background:**

Myocardial infarction (MI; location and extent of infarction) can be determined by late enhancement cardiac magnetic resonance (CMR) imaging, which requires the injection of a potentially harmful gadolinium-based contrast agent (GBCA). Alternatively, emerging research in the area of myocardial strain has shown potential to identify MI using strain values.

**Objective:**

This study aims to identify the location of MI by developing an applied algorithmic method of circumferential strain (CS) values, which are derived through a novel hierarchical template matching (HTM) method.

**Methods:**

HTM-based CS H-spread from end-diastole to end-systole was used to develop an applied method. Grid-tagging magnetic resonance imaging was used to calculate strain values in the left ventricular (LV) myocardium, followed by the 16-segment American Heart Association model. The data set was used with k-fold cross-validation to estimate the percentage reduction of H-spread among infarcted and noninfarcted LV segments. A total of 43 participants (38 MI and 5 healthy) who underwent CMR imaging were retrospectively selected. Infarcted segments detected by using this method were validated by comparison with late enhancement CMR, and the diagnostic performance of the applied algorithmic method was evaluated with a receiver operating characteristic curve test.

**Results:**

The H-spread of the CS was reduced in infarcted segments compared with noninfarcted segments of the LV. The reductions were 30% in basal segments, 30% in midventricular segments, and 20% in apical LV segments. The diagnostic accuracy of detection, using the reported method, was represented by area under the curve values, which were 0.85, 0.82, and 0.87 for basal, midventricular, and apical slices, respectively, demonstrating good agreement with the late-gadolinium enhancement–based detections.

**Conclusions:**

The proposed applied algorithmic method has the potential to accurately identify the location of infarcted LV segments without the administration of late-gadolinium enhancement. Such an approach adds the potential to safely identify MI, potentially reduce patient scanning time, and extend the utility of CMR in patients who are contraindicated for the use of GBCA.

## Introduction

### Background

Cardiovascular diseases (CVDs) account for 31% of global deaths [1]. Among CVDs, myocardial infarction (MI) can result from chronic progressive coronary atheromatous disease, with subsequent plaque rupture and thrombosis. Depending on the extent of infarction, there is potential for adverse myocardial remodeling, and subsequently, heart failure [2]. If a patient has MI, with either reduced left ventricular (LV) function or ongoing chest pain, clinicians need to evaluate ischemia and myocardial viability to determine whether there is a potential benefit to be derived from revascularization with either coronary artery bypass graft (CABG) or percutaneous coronary intervention (PCI) [2]. Ischemia can be demonstrated using cardiac magnetic resonance (CMR) imaging by assessing the first-pass perfusion, following the use of a vasodilator stress agent such as adenosine [3]. As a part of the myocardial viability test, it is crucial to show the location and extent of the infarcted myocardium [3].

### Clinical Practice and Literature Review

The current gold standard [3] for determining the location and extent of infarcted myocardium is late gadolinium enhancement (LGE), which acquires delayed CMR images following gadolinium-based contrast agent (GBCA) administration. A GBCA shows the infarcted myocardium with brighter image intensity compared with a noninfarcted myocardium [3]. However, GBCA-administered LGE has the following limitations: (1) the risk of nephrogenic systemic sclerosis in patients with advanced renal impairment [4], (2) concerns of gadolinium accumulation in tissues in normal renal patients [4], and (3) prolonged scan time resulting in adverse situations, such as panic attacks, especially in claustrophobic patients [5]. Therefore, a method that allows infarction detection, without the need for GBCA administration, has the potential to reduce patient scanning time and extend the use of CMR imaging in a wider patient population.

Strain-based techniques, such as speckle tracking and CMR-feature tracking (CMR-FT), have been previously reported to identify infarction [6-8]. Strain-based techniques do not require the use of GBCA. Hence, the aforementioned limitations of LGE could be avoided. Speckle tracking has limited accuracy [7] and CMR-FT is prone to endocardium and epicardium definition because it only uses vessel boundaries and does not include accurate details of structural deformation within the myocardium as grid-tagging magnetic resonance imaging (MRI) does [7]. The LV myocardium has a helical structure of muscles and different mechanics throughout the myocardium [9]. Therefore, the proposed method uses a novel algorithmic hierarchical template matching (HTM)–based diagnosis method [10,11], which calculates myocardium strain values by considering the details of structural deformation within the myocardium for infarction detection. The HTM method provides higher accuracy in calculating two-dimensional (2D) CMR tagging LV strain when compared with the benchmark nonrigid registration using the free-form deformation algorithm, which has been reported to be the most accurate in comparison with other state-of-the-art methods, including optical flow, harmonic phase, and B-snake grid [12,13].

### Aim of the Study

The aim of this study aims to develop an applied algorithmic method to identify MI using HTM-based circumferential strain (HTM-CS) values. The purpose of this work is to show the performance of HTM-CS-based infarction detection with respect to the gold-standard LGE-based detections.

## Methods

### Data Collection and Preparation

A data set of 38 patients with MI and 5 healthy volunteers was collected from the CMR unit of the Royal Brompton and Harefield National Health Service (NHS) Trust (RBHT) through SB. Ethical approval for retrospective data collection was obtained from the NHS (IRAS project ID: 211977). Additionally, Biomedical and Scientific Research Ethics Committee (BSREC) approval (REGO 2016–1865) was obtained from the University of Warwick to process the anonymized data.

All participants were selected retrospectively. Patients with an MI identified on CMR imaging were determined from the referral details and scan reports. The inclusion criteria were as follows: (1) a patient with a known history of infarcted myocardium, or (2) a patient referred for a clinically indicated CMR imaging scan, on the basis of symptoms suggestive of myocardial ischemia, with or without an elevation in serum troponin levels and with a confirmed myocardial infarct on the subsequent CMR imaging. Initially, 55 patients were screened for the study. Among them, 38 patients with MI were included. From the CMR referral details, these 38 patients with MI had clinical conditions such as heavy chest pain, high troponin findings, previous history of known infarction, or intervention history of CABG or PCI. Patients with nonischemic cardiomyopathy or normal findings without infarction were excluded. The characteristic details of these 38 patients with MI are summarized in Table 1. The included patients underwent a standard departmental CMR using either a vasodilator stress perfusion protocol or a viability protocol, both of which included comprehensive late gadolinium enhancement imaging. All participants with MI had anonymized images of LGE imaging and grid-tagging MRI. The images were acquired with three different 1.5T Siemens MRI scanners with ECG triggering. LGE images were acquired with sequences that allowed normal breathing of the patient and had infarcted myocardium with high-intensity values due to postgadolinium enhancement. Grid-tagging MRI was acquired with breath-holds, using a grid structure of myocardial tagging lines with a spacing of 6 mm. LGE images were not available for healthy participants.

**Table 1:** Characteristic details of the data set (N=43).

Participant types and characteristics			Value
**Patients with MI^a^ (n=38, 88%)**			
	**Sex, n (%)**		
		Male	33 (87)
		Female	5 (13)
	Age (years), mean (SD)		63.65 (13.31)
	Height (m), mean (SD)		1.74 (0.10)
	Weight (kg), mean (SD)		84.42 (19.79)
	Pixel size (mm^2^)		1.32×1.32 to 1.75×1.75
**Healthy participants (n=5, 12%)**			
	**Sex (male or female)**		
		Male	2 (40)
		Female	3 (60)
	Age (years), mean (SD)		41.20 (14.38)
	Height (m), mean (SD)		1.68 (0.08)
	Weight (kg), mean (SD)		65.2 (13.04)
	Pixel size (mm)		1.48×1.48

^a^MI: myocardial infarction.

In all the data subjects, images from 3 short-axis (SAX) planes of the LV were processed: basal, midventricular, and apical SAX planes of LV. Basal refers to the LV slice near the mitral valve and before the beginning of the papillary muscle, midventricular refers to the LV slice at the approximate middle of papillary muscle length, and apical refers to the LV slice towards the apex but above the apex. This definition is based on the literature [14,15]. The MRI SAX plane covers many anatomical details of the chest. Therefore, to efficiently process data, the LV area of each image was cropped using ImageJ [16] software. When cropping the images, we ensured that all images for a participant were coregistered well with each other and had the same image dimensions. The cropped images were processed to have normalized (zero-mean unit-variance) intensity values. The images were noise-free, that is, they did not contain imaging artifacts or motion blur artifacts.

In patients with MI, the findings of LGE imaging were used as a ground truth to differentiate between the infarcted and normal (or healthy) myocardium when an area of hyperintensity was present. LV segments were defined according to the American Heart Association (AHA) [14]. In healthy participants, all LV segments were considered healthy. The total number of infarcted segments in 38 patients with MI was 109 (basal: 38, midventricular: 44, apical: 27). The total number of healthy segments was 579 (basal: 220, midventricular: 214, apical: 145), which inlcuded 499 segments from patients with MI and 80 segments from healthy participants.

### Model of Applied HTM Method

**Figure 1:**
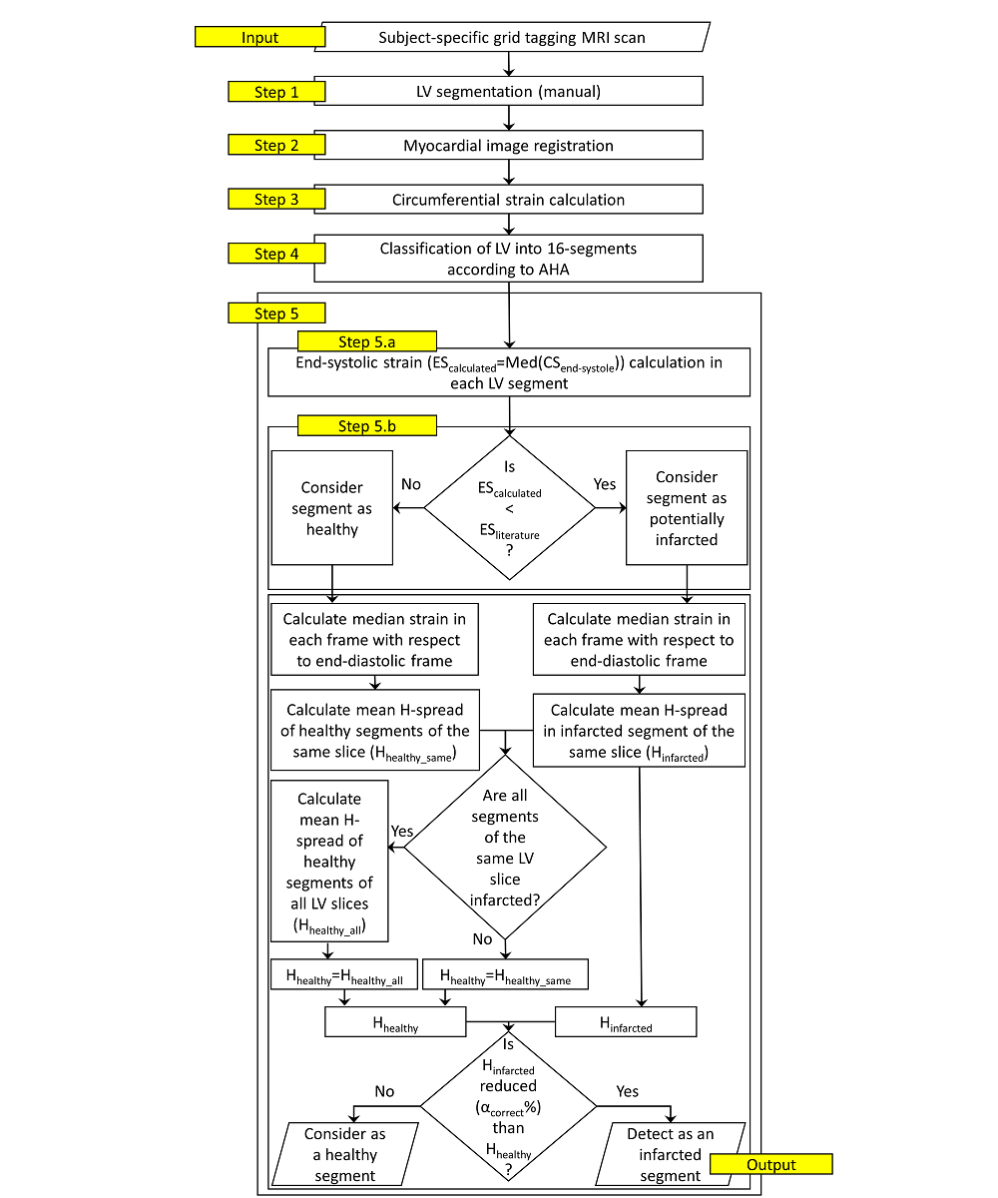
Flowchart of the proposed applied method to identify infarcted LV segments using circumferential strain values. Here, H_healthy_same_ could be H_healthy_basal_ or H_healthy_mid_ or H_healthy_apical_. HA: American Heart Association; CS: circumferential strain; ES_calculated_: median of calculated circumferential strain values at the end-systolic frame; ES_literature_: median of literature referred circumferential strain values at the end-systolic frame; LV: left ventricular or left ventricle.

The flowchart of the model is shown in Figure 1.

#### Step 1—Segmentation of LV Myocardium

The LV myocardium was manually segmented using ImageJ [16] at the end-diastolic frames of the basal, midventricular, and apical SAX planes. The segmentation was verified by a clinical expert.

#### Step 2—Myocardial Image Registration

**Figure 2:**
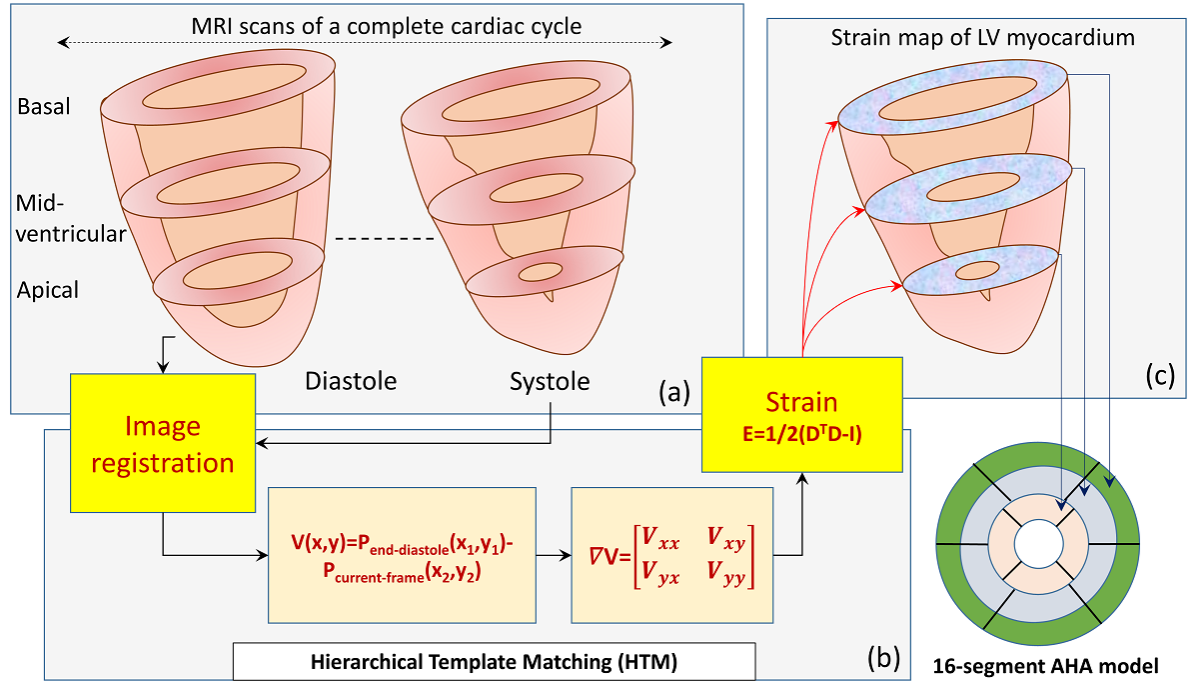
(a) MRI scans of a cardiac cycle at three LV levels: Basal, Mid-ventricular, and Apical. (b) HTM method to calculate strain values at each muscle point. V is mentioned in (Equation 2). (c) LV strain values, which are analyzed using the 16-segment AHA model. AHA: American heart association; HTM: hierarchical template matching; LV: left ventricle.

Myocardial image registration was performed using the HTM [10] method to perform myocardial tracking and strain calculation. As shown in Figure 2, HTM takes a stack of MRI with segmented myocardium as inputs. The image textures in all the images from end-diastole to end-systole were tracked using HTM. The tracking provided the position of each myocardial point during a cardiac cycle. The tracking was formulated by calculating a difference vector, *V*(*x*,*y*), between the initial position, *P_end – diastole_* (*x*_1_, *y*_1_), and current position, *P_current – frame_* (*x*_2_, *y*_2_), of a myocardial point (Equation 1). The displacement gradient at the end-systolic frame was calculated using the spatial positions at end-diastole and end-systole (Equation 2). ∇ *V* refers to the displacement gradient.

*V* (*x*, *y*) = *P_end – diastole_* (*x*_1_, *y*_1_) – *P_current – frame_* (*x*_2_, *y*_2_) (1)

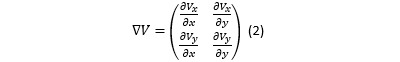



#### Step 3—Myocardial Strain Calculation

Equation 3 and Equation 4 show the deformation gradient *D* and myocardial strain (Green-Lagrange strain [17]) *E*, with respect to the end-diastolic frame, respectively, where I is the identity matrix.

*D* = (*I –* ∇ *V*)^-1^ (3)

*E* = 1/2 (*D^T^D* – *I*) (4)

Strain values at each myocardial point were calculated using a local coordinate system [18]. Only CS was used for analysis, as discussed in the Discussion section. The positive direction of CS is in the short-axis plane and counterclockwise when observed from the base. It is parallel to the surface of the epicardium and perpendicular to the long axis of the LV.

#### Step 4—Classification of LV into 16-Segments

The LV myocardium was divided into 16 segments according to the 16-segment AHA model [14]. The basal, midventricular, and apical slices were divided into six, six, and four segments, respectively. Thereafter, the CS in each segment was used during Step 5 to separate healthy and infarcted segments.

#### Step 5—Detection of Infarcted Segments

##### Initial Detection Using End-Systolic HTM-CS

The median end-systolic strain in each of the AHA LV segments was calculated as *ES_calculated_* = *Med*(*CS_end – systole_*). Here, a median of strain values was used to avoid the influence of any large outliers. Subsequently, the calculated median strain was compared with the literature benchmark CS values (*ES_literature_*) for healthy segments of LV, as mentioned in the literature [18] (Table 2). If the *Med*(*CS_end-systole_*) value was less than the benchmark CS values of the healthy myocardium, the segment was considered as a “potentially infarcted” segment (Figure 1).

**Table 2:** End-systolic circumferential strain in healthy left ventricular myocardium from literature.

Left ventricular slice	Anteroseptal strain, mean (SD)	Inferoseptal strain, mean (SD)	Anterior strain, mean (SD)	Anterolateral strain, mean (SD)	Inferolateral strain, mean (SD)	Inferior strain, mean (SD)
Basal	−0.17 (0.03)	−0.17 (0.03)	−0.20 (0.03)	−0.21 (0.03)	−0.21 (0.03)	−0.16 (0.03)
Midventricular	−0.16 (0.03)	−0.16 (0.03)	−0.23 (0.04)	−0.22 (0.03)	−0.22 (0.03)	−0.16 (0.05)
Apical	−0.18 (0.03)	−0.18 (0.03)	−0.24 (0.06)	−0.24 (0.04)	−0.24 (0.04)	−0.23 (0.04)

##### Final Detection Using H-spread of HTM-CS

Infarcted myocardium does not contract or lengthen like healthy muscles because of the presence of fibrosis [19]. This property was quantified by the H-spread of the HTM-CS distribution.

**Figure 3:**
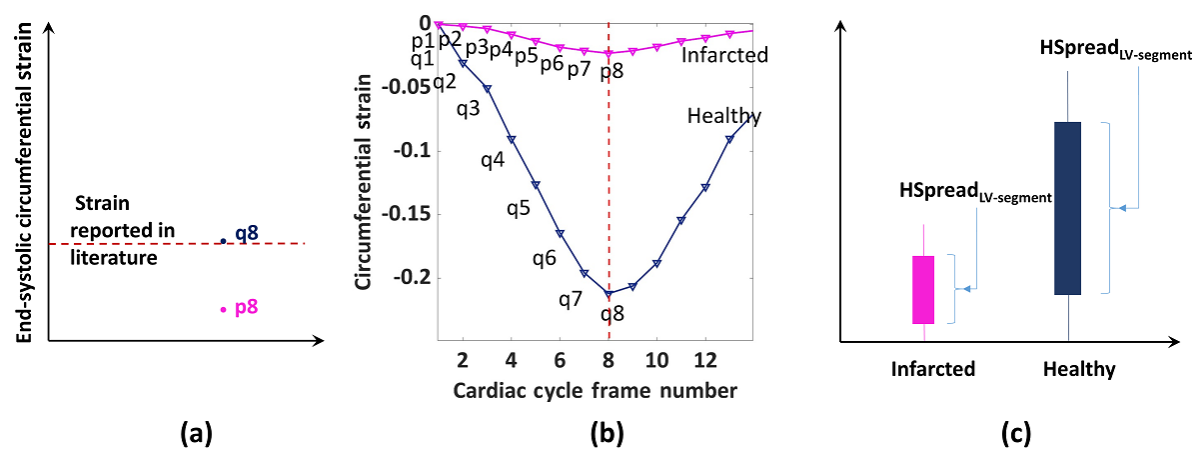
Representation of an infarcted and a healthy segment using circumferential strain. (a) End-systolic strain in a healthy segment (q8) and an infarcted segment (p8). (b) pi and qi represent median strain values at the ith frame of a cardiac cycle. (c) Strain HSpread is calculated using (Equation 5).

H-Spread of the HTM-CS values in each AHA segment was calculated by the union of median strain values in each frame from end-diastole to end-systole. For example, the H-spread of an infarcted segment (Figure 3; Equation 5) was calculated by the union of values p1,p2,…,p8 (Figure 3). Similarly, the H-spread of the healthy segment was calculated by the union of values q1,q2,…,q8 (Figure 3). The MATLAB function iqr() was used to calculate the H-spread [20].



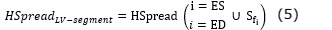



where ES = end-systolic frame, ED = end-diastolic frame, and S_fi_ = *Med*(*CS_end – systole_*) at the *i*^th^ frame.

After calculating the H-spread of “potentially infarcted” segments, *“Infarction Condition”* was checked to decide whether the “potentially infarcted” segment was actually an infarcted segment; during this step, the H-spread values were also calculated, as described in Equation 6.

*H_infarcted_* = *HSpread_LV – segment_*

*H_healthy_basal_* = *µ_basal_*(*HSpread_LV – segment_*)

*H_healthy_mid_* = *µ_mid_*(*HSpread_LV – segment_*)

*H_healthy_apical_* = *µ_apical_*(*HSpread_LV – segment_*)

H_healthy_all_ = µ_basal–mid–apical_(HSpread_LV – segment_)

*H_healthy_* = *H_healthy_basal_ OR H_healthy_mid_ OR H_healthy_apical_ OR H_healthy_all_* (6)

##### Infarction Condition

A potentially infarcted segment has at least α% reduced strain H-spread compared to the mean H-spread of remaining LV segments of the same LV slice. If all segments of a slice are infarcted, then healthy segments of the whole LV are considered instead of only considering the same slice.

α could be any value greater than 0 and less than 100. For example, assume that segments 1 and 2 of a basal slice are “potentially infarcted” segments. These segments will be considered as actually infarcted if *HSpread_LV – segment_* is at least α% reduced compared with the mean H-spread of other segments in the basal slice (*H_healthy_basal_*), which includes segments 3, 4, 5, and 6. When all segments of a slice were “potentially infarcted,” healthy segments of basal, midventricular, and apical slices were used to calculate (*H_healthy_all_*). For example, if segments 13, 14, 15, and 16 of an apical slice were “potentially infarcted,” the healthy segments of basal and midventricular slices were considered together during H-spread comparison of the “Infarction Condition.”

In this work, the following values of α were considered (Equation 7) to determine the most appropriate α_correct_ value, and it was assumed that the values within this range of α values would not change the results considerably.

α = {10, 20, 30, …, 100} (7)

The detection accuracy using different α values is discussed in the Results section. The α value corresponding to the highest accuracy was called α_correct_, which was then used to detect the MI. Tests were performed using k-fold cross-validation [21,22].

### K-Fold Cross-Validation for Model Training and Testing

In each patient, infarcted segments (AHA segments) were identified using LGE CMR imaging by a cardiothoracic consultant and surgeon who had more than 10 years of experience. These infarcted LV segments were used as the ground truth to validate the proposed HTM-CS–based predictions. Therefore, the validation hypothesis for the statistical analysis was that the infarcted LV myocardial AHA segments which were identified using HTM-CS would be the same as the ground truth of LGE. This section explains k-fold cross-validation [22] using the receiver operating characteristics (ROC) curve test [23,24], which was used in this study to validate the proposed method.

The area under the curve (AUC) of the true negative rate (sensitivity) and the false-positive rate (1-specificity) were calculated from ROC curve tests [23,24], as prediction performance criteria, where AUC 1.0 is the highest accuracy and AUC 0.5 is the lowest accuracy. ROC tests were performed using the MATLAB function perfcurve() [25]. The ROC tests had a 95% CI. Data were prepared by dividing each LV into 16 AHA segments, and the segments were arranged as per basal, midventricular, and apical slices. The total number of basal segments was 258 (258 = 43 × 6), the total number of midventricular segments was 258 (258 = 43 × 6), and the total number of apical segments was 172 (172 = 43 × 4). Each segment was assigned a label, as infarcted or healthy, according to the LGE ground truth. Then, HTM-CS H-spread reduction was assigned to each “potentially infarcted” segment (healthy segments were considered with 0% H-spread reduction).

During the k-fold tests, *“Infarction Condition”* was evaluated using each α value of Equation 7. Each test assigned a score to each segment as infarcted or healthy. For example, an evaluation test with α=10 scored a potentially infarcted LV segment as infarcted if it satisfied the *“Infarction Condition*;*”* otherwise, it was scored as healthy. These scores and the ground truth labels of each segment were given as input to the ROC test, as mentioned previously. The α value was selected as α_correct_ if the corresponding ROC test had the highest AUC. This α_correct_ value was used with the test data set during the k-fold test to calculate the final accuracy.

**Figure 4:**
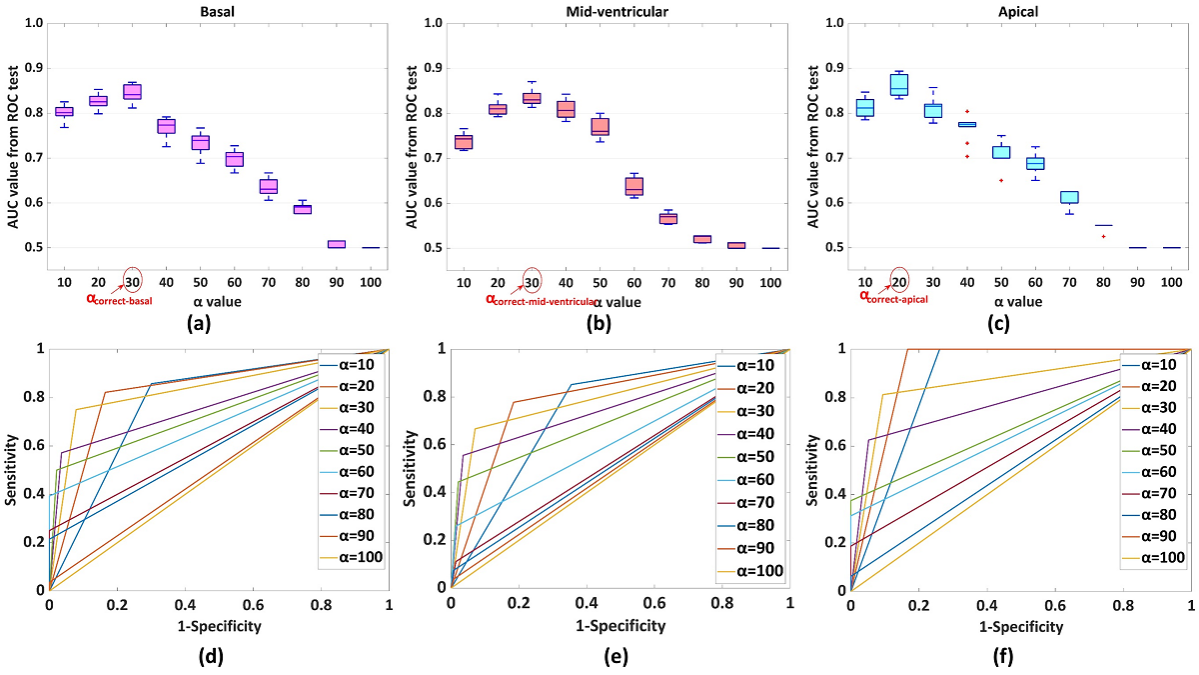
Overall results of 10 k-fold cross-validation tests. Overall AUC values using each α are shown in (a) basal, (b) mid-ventricular, and (c) apical slices. Red circles denote α_correct_ corresponding to the maximum AUC for the respective slice. Examples of ROC test results for (d) basal, (e) mid-ventricular, and (f) apical slices, respectively. AUC: area under the curve; ROC: receiver operating characteristic.

The training data set and test data set were split as per k-fold cross-validation tests. Initially, k=5 and k=10 were considered, as suggested in the literature [22]. After that, k-fold tests were performed with k=10. When the experiments were performed with the test data set using k=10, we noted that the results were less realistic in the case of k=10. For example, for k=10 using test data, the ROC tests had an AUC of 1.0 (ie, 100% accuracy) in 3 tests (1 out of 10 in each of basal, midventricular, and apical LV slices) and AUC of 0.5 (ie, 0% accuracy) in 1 test (1 out of 10 in apical slices). However, an AUC of 1.0 and 0.5 were not found in the case of k=5. Therefore, we used k=5, and the k-fold tests were repeated 10 times in each basal, midventricular, and apical LV slice with a random selection of data. Figure 4 shows the analysis with k=5.

Using the test data set, true positives (infarcted segments detected as infarcted), true negatives (noninfarcted segments detected as noninfarcted), false positives (noninfarcted segments detected as infarcted), and false negatives (infarcted segments detected as noninfarcted) were calculated. Additionally, the sensitivity (true positive rate) and 1-specificity (false negative rate) of the detection of infarcted segments are provided.

## Results

### Primary Analysis of the Model Using Training Data

To find α_correct_ among α values, a training data set of k-fold cross-validation test was used. Figure 4 presents the AUC values in each ROC test for the basal, midventricular, and apical slices using the training data set. α_correct_ was selected from the ROC test, corresponding to the maximum AUC. Accordingly, α_correct-basal_, α_correct-midventricular_, and α_correct-apical_ were found. Figure 4 shows the maximum AUC at α=30, midventricular at α=30, and apical at α=20. Therefore, α_correct-basal_=30, α_correct-midventricular_=30, and α_correct-apical_=20. Further, k-fold cross-validation tests were performed with test data and α_correct_ values.

### Accuracy Analysis of HTM-CS-Based Model Using Test Data

**Figure 5:**
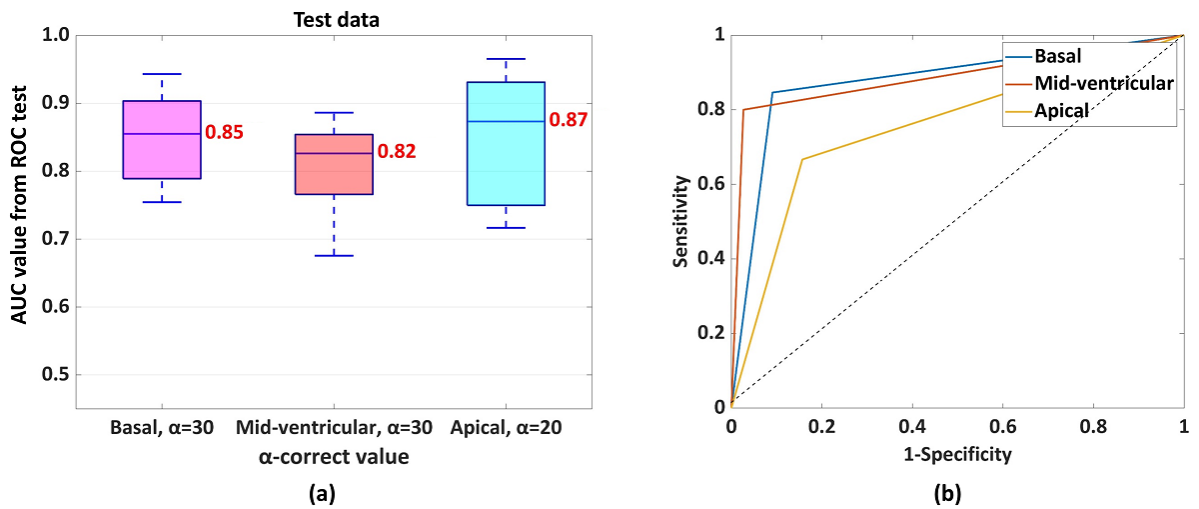
(a) Average of 10 k-fold cross-validation tests. Accuracy of detecting infarcted segments using the proposed method with α_correct_ in basal, mid-ventricular, and apical slices using test data from the k-fold cross-validation tests. (b) An example of an ROC test result using the test data. AUC: area under the curve; ROC: receiver operating characteristic.

To analyze the performance of the HTM-CS-based method, the test data set was used. Each -fold cross-validation test used a random 5% sample as test data. α_correct-basal_=30, α_correct-midventricular_=30, and α_correct-apical_=20 were considered with “Infarction Condition” to predict each segment as healthy or infarcted. The results are plotted in Figure 5. In Figure 5, the results of the ROC tests were derived using the MATLAB function perfcurve() [25]. The basal, midventricular, and apical areas had AUC values of 0.85, 0.82, and 0.87, respectively. Table 3 shows an example of a detection.

**Table 3:** Analysis of a myocardial infarction patient. Truth and Detected show the results of late gadolinium enhancement and the proposed method, respectively.

Patient number		Truth		Detected	
		Infarcted?	Which segment?	Infarcted?	Which segment?
**Patient number D9**					
	Basal	Yes	1,2	Yes	1,2
	Midventricular	Yes	7,8	Yes	7,8,12
	Apical	Yes	13,14	Yes	13,14,15

“Truth” shows infarcted segments using LGE and “Detected” shows infarcted segments using the proposed method. The best case would be to have the same “Truth” and “Detected” segments in all three slices. Table 4 summarizes the detections in test patients.

The higher true-positive rate and the lower false-positive rate together determine the best result. For detections in a patient with MI, the best case should have true-positive rate=1 and false-positive rate=0, and in a healthy participant, false-positive rate=0 should be the best case.

**Figure 6:**
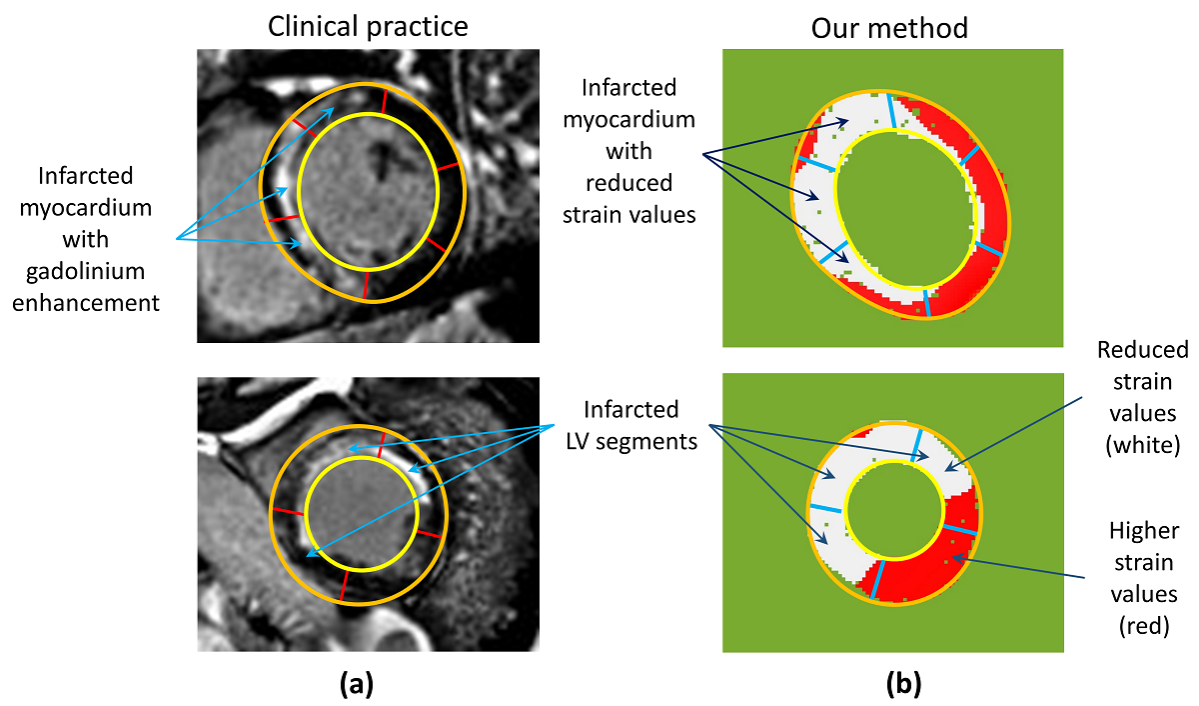
(a) LGE showing infarcted segments in white; (b) proposed CS-based analysis showing infarcted and healthy LV segments in white and red, respectively. CS: circumferential strain; HTM: hierarchical template matching; LGE: late gadolinium enhancement.

Figure 6 shows an example of detecting infarcted LV segments using the proposed algorithmic method compared with the LGE method. As shown in the LGE images, the white area of the LV myocardium (due to gadolinium deposition) highlights infarction. In strain analysis, red color shows healthy LV segments and white color shows infarcted LV segments. Some of the segments are both red and white. All segments were characterized as healthy or infarcted by considering the H-spread as mentioned in the “Infarction Condition” of the Methods section.

**Table 4:** Results of detecting infarcted left ventricular segments.

Patient number	Total LV^a^ segments	Infarcted segments	TP^b^	TN^c^	FP^d^	FN^e^	Sensitivity (TPR)^f^ = TP/(TP + FN)	1-Specificity (FPR)^g^ = FP/(TN + FP)^h^
D1^i^	16	5	3	9	2	2	0.75	0.18
D2	16	2	1	13	1	1	0.5	0.07
D3	16	1	1	12	3	0	0.5	0.2
D4	16	1	1	14	1	0	1	0.06
D5	16	2	2	14	0	0	0.25	0
D6	16	3	3	12	1	0	1	0.07
D7	16	7	5	9	0	2	1	0
D8	16	6	6	8	2	0	0.66	0.2
D9	16	5	5	11	0	0	1	0
H1^j^	16	0	0	12	4	0	NaN^k^	0.25
H2	16	0	0	16	0	0	NaN	0

^a^LV: left ventricular.

^b^TP: true positives.

^c^TN: true negatives.

^d^FP: false positives.

^e^FN: false negatives.

^f^TPR: true-positive rate (sensitivity).

^g^FPR: false-positive rate (1-specificity).

^h^The higher sensitivity and the lower (1-specificity) together determines the best result (eg, D7, D9, and H2).

^i^D: diseased.

^j^H: healthy.

^k^NaN: not a number.

## Discussion

### Principal Findings

This paper elaborates an applied HTM-CS–based infarction detection method, which does not require GBCA administration for conventional late gadolinium CMR imaging. The results demonstrated the promising accuracy of the proposed method, which was compared with the gold-standard method of infarction detection, that is, LGE.

### Data Set

The data set used in this study is a grid-tagging short-axis MRI because the scope of the article is to show that the CS-based applied method can identify MI. This CS can be calculated using grid-tagging CMR images, as reported in the literature [26].

### Model of the Applied HTM Method

In the proposed algorithmic model, the HTM method is used for myocardial tracking. HTM uses normalized cross-correlation in a hierarchical manner to establish point correlations. In this hierarchical matching process, as defined in the article [10], the larger features of images (ie, templates) can correlate well with larger areas of images; however, they are not efficient in matching smaller areas of images. Therefore, HTM has also used smaller image features (ie, segments, chunks, and windows [10]) to correlate smaller areas of images and provide a dense set of correlated points among images. This hierarchical matching is ultimately helpful for myocardial tracking, which is also effective in smaller areas of LV images.

We used CS because HTM uses grid-tagged CMR images in an image texture tracking-based method, and a sequence of grid-tagged CMR images can show better circumferential movement compared with radial movement. A similar observation is reported in the literature [26], with a potential solution to merge cine CMR images with grid-tagged CMR images to capture and use radial movement more efficiently. However, the scope of this paper is limited to showing the applicability of CS; therefore, we have used only CS.

In Step 5a, the benchmark strain values of Table 2 were obtained from the literature [18]. Table 2 reported strain values for LV septal and lateral areas without subdividing it into inferoseptal, anteroseptal, inferolateral, and anterolateral segments. Therefore, to be able to use literature-referred values, we used the same strain values for both anterolateral and inferolateral segments, and similarly, the same strain values for the anteroseptal and inferoseptal segments.

During Step 5b, the “Infarction Condition” is evaluated, which compares the strain H-spread among the segments of the same LV slice, and in specific conditions, when all segments of an LV slice are infarcted, the healthy segments of other slices are used for H-spread comparison. The clarification is that, in all 43 participants, the overall mean of only healthy basal segments (µ(H_healthy_basal_)), an overall mean of only midventricular healthy segments (µ(H_healthy_mid_)), and an overall mean of only apical healthy segments (µ(H_healthy_apical_)) were mean 0.0772 (SD 0.0372), 0.0862 (SD 0.0366), and 0.0992 (SD 0.0491), respectively. Moreover, the mean H-spread of all healthy segments from all slices together (µ(H_healthy_all_)) was 0.0851 (SD 0.0271). Here, we performed a paired-sample t-test, which hypothesized that the distribution of differences between each pair of H_healthy_all_ and H_healthy_apical_ (or H_healthy_basal_ or H_healthy_mid_) is a normal distribution with mean zero and unknown variance. The test does not reject our hypothesis for *P*=.04. Therefore, we assumed that such differences among them will not change the accuracy of the method considerably, and therefore, for specific conditions when all segments of a slice are infarcted, we have used H_healthy_all_ during comparison, instead of using only H_healthy_basal_ or H_healthy_mid_ or H_healthy_apical_.

In k-fold cross-validation, the results are derived using k=5 because k=10 has reported a high variance in the prediction (some of the tests have 100% prediction accuracy and some of them have approximately 50%). Therefore, k=5 was used for consistent prediction, as suggested in the literature [22].

### Validation Method

Validation was performed using LGE CMR images, which is a clinical gold standard method for identifying infarction using CMR imaging [3].

### Accuracy of the Applied HTM Method

The infarcted myocardium does not shorten or lengthen the healthy myocardium due to replacement fibrosis, and the average shortening of healthy basal muscles in the circumferential direction is 20% [18,27]. Moreover, due to nonuniform cardiac LV mechanics, basal, midventricular, and apical slices have different average end-systolic shortening of 18.5%, 19.25%, and 22.25%, respectively [18]. Therefore, the proposed method has analyzed detections separately in basal, midventricular, and apical slices to find separate α_correct-basal_, α_correct-midventricular_, and α_correct-apical_. α_correct-basal_=30 and α_correct-midventricular_=30 show that the infarcted LV segments have at least 30% reduced strain H-spread compared with the healthy LV segments in basal and midventricular slices, respectively. Similarly, α_correct-apical_=20 shows that the infarcted LV segments in apical slices have at least 20% reduced strain H-spread compared with healthy LV segments. This difference is due to partially infarcted LV segments. Figure 5 shows different accuracies at different LV levels. Moreover, Table 4 shows that some of the infarcted segments were detected as healthy (false negatives). A possible reason is that the proposed method is fundamentally based on image texture tracking and is sensitive to image quality. LV slices suffer from texture fading due to breathing or blood flow, and motion artifacts due to patient movement. Consequently, the method could not track muscles, which cause an error in strain calculation and ultimately result in incorrect detection. Table 4 shows the results of randomly selected 9 patients with MI and 2 healthy participants. Healthy volunteers do not have infarcted segments. Therefore, true positive and false negative detections were zero, and the true-positive rate was not a number. However, there were false detections in healthy participants, which resulted in a false-positive rate. False positives were due to reduced CS values.

### Clinical Impact

The proposed method could detect infarcted LV segments without using GBCA, which can extend the utility of CMR in conditions such as chronic kidney disease stage 4 or 5 patients (glomerular filtration rate <30 ml/min/1.73 m^2^ [28]). These patients have a contraindication to the use of GBCA due to the risk of nephrogenic systemic fibrosis. Moreover, the concerns of gadolinium accumulation in normal renal patients can be avoided with the proposed method. The method could potentially reduce scanning time, as it identifies infarction by postimage analysis, and a patient is not required to be inside the scanner for an additional LGE scan, which in most instances requires at least 5 to 10 min extra scanning time following GBCA administration. A study reported that patients who undergo MRI often have claustrophobia, anxiety, and panic attacks (approximately 13%) [5]. Therefore, an overall reduced scanning time may help improve patient care. Moreover, GBCA usage costs an additional €50 (US $61.39) to a patient [29]. Hence, HTM-CS–based analysis could be more economical.

### Future Work and Limitations

The proposed algorithmic method used three 2D slices and a 16-segment AHA model. However, the methodology could adapt to a different number of segments. A higher number of slices could be included after a rigorous literature review to obtain generalized strain values. Hypokinetic segments could be detected as infarcted using the proposed method. The proposed method can locate infarcted LV segments, and further investigations are required to determine the extent of infarction (transmurality). A possible reason is the lower resolution of grid-tagging MRI and faded endocardium and epicardium borders. However, the method could be improved by combining multiple CMR imaging modalities for higher accuracy. The method is not fully automatic; therefore, evaluation at the scanner is not possible at this stage. As the method is semiautomatic and requires image cropping, manual segmentation, and nonrigid image registration, the evaluation time is subjective, such as 5 to 6 hours for a patient. Moreover, images with artifacts due to breath-holding, blood flow, or motion could affect the accuracy of our method.

### Conclusions

In this paper, an applied method for detecting MI based on CS analysis is proposed. The results are compared with the clinical gold-standard (LGE) in detecting MI, and it is observed that the proposed HTM-CS–based approach can provide accurate detections. Moreover, the proposed method avoids the use of GBCA, leading to reduced material cost and scanning time, which may be of particular benefit in individuals with claustrophobia.

## Abbreviations

2D: two-dimensional
AHA: American Heart Association
AUC: area under the curve
CABG: coronary artery bypass graft
CMR: cardiac magnetic resonance
CMR-FT: CMR-feature tracking
CS: circumferential strain
CVD: cardiovascular diseases
GBCA: gadolinium-based contrast agent
HDR: Health Data Research
HTM: hierarchical template matching
HTM-CS: HTM-based CS
LGE: late gadolinium enhancement
LV: left ventricular or left ventricle
MI: myocardial infarction
MRI: magnetic resonance imaging
NHS: National Health Service
PCI: percutaneous coronary intervention
RBHT: Royal Brompton and Harefield NHS Foundation Trust
ROC: receiver operating characteristics
SAX: short-axis
